# Home- and Community-Based Service Use and Preferences Among Post-9/11 Veterans With or at High Risk of Alzheimer Disease and Related Dementia and Their Caregivers: Protocol for a Mixed Methods Observational Study

**DOI:** 10.2196/83629

**Published:** 2026-04-29

**Authors:** Erin D Bouldin, Amanda Cheney, Mary Jo Pugh, Kayla Morales, Roxana E Delgado

**Affiliations:** 1 Informatics, Decision-Enhancement and Analytic Sciences Center, Health Systems Research, Department of Veterans Affairs Medical Center, 500 Foothill Blvd, Salt Lake City, UT, 84148, United States, 1 8012133249; 2 Division of Epidemiology, Department of Internal Medicine, University of Utah Spencer Fox Eccles School of Medicine, Salt Lake City, UT, United States; 3 Caring for the Caregiver Program, School of Nursing, UT Health San Antonio, San Antonio, TX, United States

**Keywords:** veterans, frontotemporal dementia, early-onset dementia, traumatic brain injury, home- and community-based services, personal health services, patient preference, independent living, observational study

## Abstract

**Background:**

Veterans have an increased risk of developing Alzheimer disease and related dementia (ADRD) due to military exposures such as traumatic brain injury. There is a lack of information on home- and community-based services (HCBS) use among Veterans who served in the post-9/11 era and their caregivers.

**Objective:**

This study aims to (1) quantify HCBS use among post-9/11 Veterans with or at higher risk of ADRD, (2) identify facilitators, barriers, and preferences for HCBS among Veterans and family caregivers, and (3) prioritize HCBS interventions with input from Veterans and family caregivers.

**Methods:**

This study will include post-9/11 Veterans aged 65 years or younger with early-onset Alzheimer disease or frontotemporal dementia (current ADRD), and Veterans at elevated ADRD risk due to traumatic brain injury or cognitive dysfunction. Veterans’ family caregivers will also be recruited. Secondary data will come from the Department of Veterans Affairs (VA), the Department of Defense, and a previous neurotrauma study. Using VA data augmented with Centers for Medicare and Medicaid Services data, we will characterize HCBS utilization. To address aim 1, we will calculate the crude and adjusted cumulative frequency of HCBS use and the proportion of Veterans using a service among Veterans with ADRD, and those at higher and lower risk for ADRD. We will compare groups using *t* tests for continuous measures (number of services) and chi-square tests for categorical measures (any service use). To address aim 2, we will interview Veterans and caregivers to identify facilitators and barriers to HCBS use. We will use descriptive content analysis, including rich descriptions, coding, and theme identification. Finally, to address aim 3, we will use a modified Delphi approach to identify and rank HCBS modifications that would increase use. Using the ranking data, we will consider items to have consensus on high importance if 70% or more respond “important” or “very important.” Participants for primary data collection will be recruited from prior studies, VA health systems data, VA clinics, and Veteran- and caregiver-serving organizations.

**Results:**

This study was reviewed by the institutional review boards of the University of Utah, Salt Lake City Veterans Affairs, and UT Health at San Antonio and classified as exempt. The 46,053 Veterans in the preliminary aim 1 cohort (903 with early-onset Alzheimer disease/frontotemporal dementia and 45,150 at-risk Veterans matched on age and index year) averaged 55 years old at the index date and were mostly male (38,842/46,053, 84%) and non-Hispanic White (28,016/46,053, 61%).

**Conclusions:**

This study will quantify current HCBS use and identify barriers and needs of Veterans with or at higher risk of ADRD and their caregivers. It will identify HCBS modifications that have consensus for needed changes, which will be shared with health system leaders.

## Introduction

### Background

The National Institutes of Health summit on Alzheimer Disease and Related Disorders highlighted that both Alzheimer disease and frontotemporal dementia (FTD) have significant lifelong consequences that are amplified in those with early-onset dementia (EOD) [1]. Veterans are at an increased risk of developing Alzheimer disease and related dementias (ADRD) as a result of exposures, including traumatic brain injury (TBI) [2-6]. ADRD substantially impacts the quality of life for Veterans experiencing it and their family and friends who help care for them (herein “caregivers”) [7-9]. Caregivers of Veterans with ADRD often experience poorer health outcomes in their daily lives, including depression, anxiety, and physical injuries, along with financial burden, social isolation, and significantly long and demanding hours of care [10-13].

Having a caregiver can help assure the health and quality of life of people with long-term health conditions and disability, but caregivers need support to maintain their own health and fulfill their role, and this support needs to be tailored to their situation and needs. A broad array of services and supports is available outside of traditional health care settings to enable community living, known collectively as home- and community-based services (HCBS) [14]. HCBS may be used by Veterans to provide targeted, short-term assistance like home care following a hospitalization or long-term assistance with household management. Common types of HCBS include home health care, which may entail the Veteran receiving specific care like wound care from a nurse or more general house-keeping services; adult day programs, which are typically held at a community center and have organized activities and supervision for people living with various types of disability; and respite programs, which provide in-home helpers who can stay with a Veteran while the caregiver takes time to rest or complete other tasks. Veterans Affairs (VA) offers a number of HCBS programs, including homemaker-home health aide, Veteran-directed care, home-based primary care, and respite care [15]. Additionally, Veterans may be eligible for non-VA HCBS through Medicaid or local community agencies or organizations.

The vast majority of caregivers for Veterans—about 10.5 million of the 14.3 million (74%)—are caring for a Veteran who is older than 60 years [16]. Similarly, most (70%) VA-paid or VA-delivered HCBS are used by Veterans aged 65 years or older [17]. Structurally, HCBS are administered by Geriatrics and Extended Care within the VA. Given that older adults have a higher prevalence of disability and care needs, it is reasonable that HCBS has primarily been tested in and designed for this segment of the population and that HCBS services are administratively centered alongside other services for older Veterans. But the need for HCBS among younger Veterans is substantial; it is higher than among their civilian counterparts because of injuries like TBI [9]. Also, these Veterans’ caregivers are, on average, younger and thus may have different needs and preferences for services than older caregivers, given their different developmental and life stages. These factors must be considered to develop effective and acceptable HCBS for post-9/11 veterans with EOD.

According to the US Census in 2019, there are 3.8 million post-9/11 Veterans, defined as US military service members who were active on September 12, 2001 or later [18]. Veterans are at heightened risk of developing Alzheimer disease (AD)/ADRD as a result of exposures including TBI and posttraumatic stress disorder [2-6]. Of the post-9/11 deployed Veterans, 11% to 23% experienced TBI, most of which were mild TBIs [19]. Furthermore, having multiple conditions appears to increase the cumulative risk of AD/ADRD development [20,21], and once someone develops AD/ADRD, a history of TBI may lead to more rapid impairments [22]. Because post-9/11 Veterans are more racially and ethnically diverse than older Veterans [23] and the incidence of AD/ADRD is higher among people who are African-American or Hispanic [24], there may be more cases of dementia among Veterans in the future than now. Taken together, the current evidence demonstrates that AD/ADRD is critical to plan for and, if possible, prevent among post-9/11 Veterans.

Previous studies revealed that tailored HCBS options are needed to fill unmet needs for Veterans with ADRD [25], but those studies did not address the issue of EOD, which data suggest is a significant emerging trend [26]. The post-9/11 cohort of Veterans is, on average, younger than 65 years, and therefore, EOD is of greater relevance. A previous study developed an algorithm to identify ADRD in this group of Veterans and found that the positive predictive value of diagnosis codes for EOD and FTD in administrative data was 0.88‐0.96 [26,27]. For other types of ADRD, the diagnosis codes had lower positive predictive values, suggesting it is more difficult to identify dementia types other than EOD and FTD in these younger Veterans. Studies of HCBS among people with ADRD and other conditions have identified racial and ethnic disparities in HCBS use [28-30]. Among veterans with EOD, there also may be unique needs and preferences among those from different racial and ethnic backgrounds [30,31] and those living in rural areas [32-34].

Expanding access to HCBS for Veterans could potentially reduce institutionalization and the costs associated with it while improving quality of life among Veterans and their caregivers, honoring Veterans’ preferences to remain at home, and achieving health equity. Although the literature is mixed on the impact of HCBS on institutionalization and costs, especially among older adults [31,35], existing evidence and our own work suggest that HCBS could reduce caregiver burden and improve quality of life for post-9/11 Veterans with TBI, EOD, and their caregivers. While caregivers of people with ADRD are a well-studied population overall, those caring for someone with ADRD subsequent to TBI and other experiences common among Veterans have received less attention. Likewise, little research has focused on Veterans with EOD and sought to identify their needs and preferences—both present and projected—along with those of their caregivers.

### Hypothesis and Objectives

The aims of this study are to (1) quantify HCBS use among post-9/11 Veterans with or at higher risk of ADRD, (2) identify facilitators, barriers, and preferences for HCBS among these Veterans and their family caregivers, and (3) prioritize HCBS interventions with input from Veterans and their caregivers. Based on published literature and our preliminary studies, we hypothesize that post-9/11 Veterans with early-onset AD/FTD or at higher risk of ADRD and their caregivers use HCBS with greater frequency than their peers at lower risk of ADRD. We also expect HCBS use to be less common in this cohort than among older Veterans with ADRD. We further hypothesize that Veterans and caregivers in our study will report unique barriers to and preferences for accessing and using HCBS compared with those of older adults with ADRD or non-Veterans. Therefore, we expect that based on feedback from Veterans and their caregivers, we will identify novel strategies to meet the HCBS needs of post-9/11 Veterans with early-onset AD/FTD or at higher risk of ADRD and their caregivers.

## Methods

### Conceptual Framework

**Figure 1.**
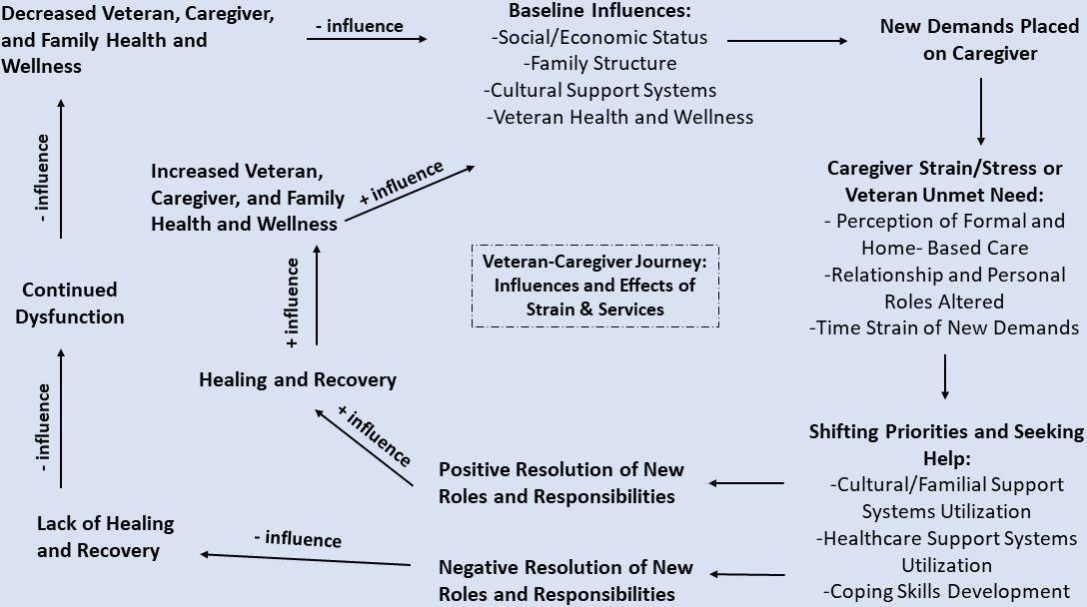
Conceptual framework, incorporating Raina’s theoretical framework, MVC journey map, and Andersen model of behavioral health [36-38]. MVC: Military and Veteran Caregiver.

Our study is guided by a theoretical framework based on the experiences of Veterans and their caregivers. This theoretical framework (Figure 1) incorporates Raina’s conceptual model of the caregiving process and caregiver burden [36] along with the Military and Veteran Caregiver Journey Map developed by the Elizabeth Dole Foundation in partnership with the VA and Philips [37]. It also includes aspects of the Andersen and Newman Model of Behavioral Health to describe health services utilization [38]. Our theoretical framework, therefore, posits that personal characteristics, family structure, and health status of Veterans and caregivers, along with the changing demands caregivers experience over time in the caregiving role, create caregiver strain and unmet needs for Veterans. Either of these 2 experiences—that is, caregiver strain or Veteran unmet needs—can result in seeking additional help, including using HCBS. Depending on whether that additional help is accessible and meets the caregiver and Veteran needs, they may experience positive or negative effects on their health and well-being. In this study, we are using the conceptual model to inform the statistical models we will use to evaluate differences in HCBS use. We have identified content from the Baseline Influences portion of the model that is available within the dataset and potentially explains some of the variation in service use. Additionally, we are using the model to develop the interview guides by asking about components of Caregiver Strain/Stress and Veteran Unmet Need and Shifting Priorities and Seeking Help so that we can understand how these experiential and health system factors impact HCBS use and preferences.

### Study Design

**Figure 2.**
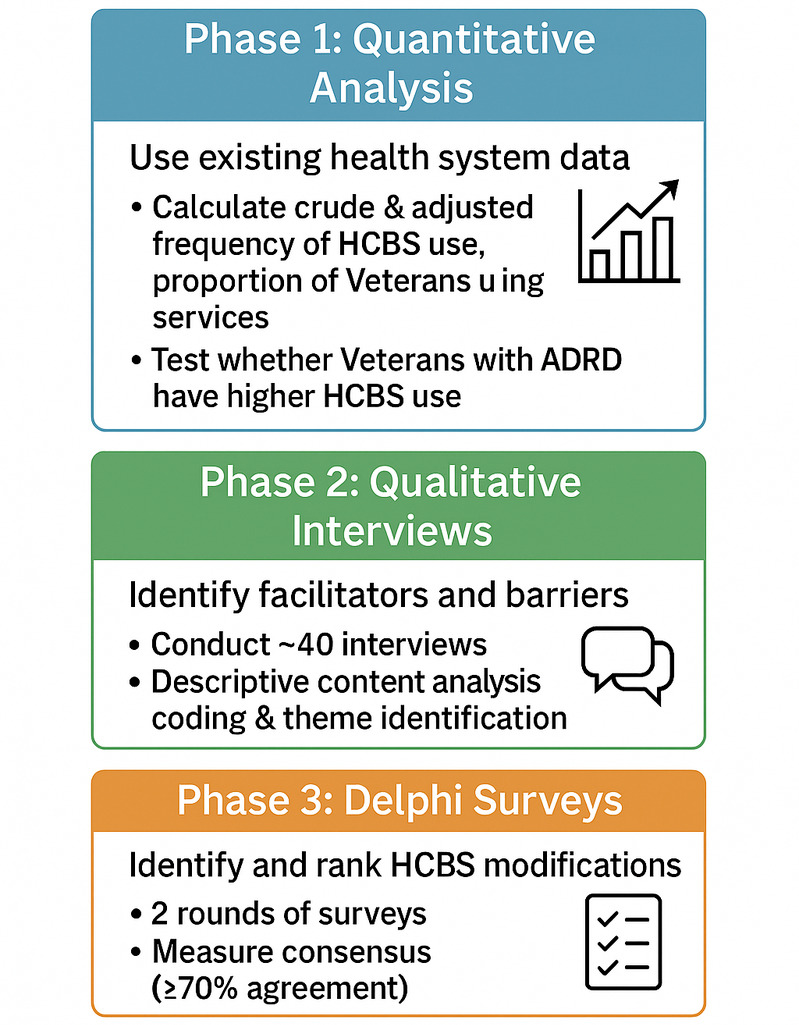
Summary of study activities for each aim (phase). ADRD: Alzheimer disease and related dementia; HCBS: home- and community-based service.

In this sequential mixed methods observational study, we will conduct a retrospective analysis of HCBS utilization; a qualitative study of facilitators, barriers, and preferences related to HCBS use and design; and a modified Delphi survey among 2 groups of post-9/11 Veterans and their caregivers: those with early-onset AD/FTD or at higher risk of ADRD and those at lower risk of ADRD (Figure 2). We will leverage existing data from previous studies for the retrospective component. For primary data collection, we will recruit participants who agreed to be re-contacted from existing data and previous and ongoing studies of post-traumatic epilepsy, TBI, and Military and Veteran Caregiver outcomes conducted by the study team for the qualitative components, supplemented with additional recruitment strategies as needed. Whenever possible in analyses based on sample size, we will distinguish Veterans with early-onset AD/FTD from those at higher risk of ADRD to identify any differences between these 2 groups.

### Study Population

For retrospective analyses, we will identify Veterans using data from the Characterizing Health Outcomes in post-9/11 Veterans (CHOP) repository, which is the foundation for the Long-term Impact of Military Relevant Brain Injury Consortium (LIMBIC) Phenotype study. The LIMBIC dataset includes a longitudinal Department of Defense (DOD) and VA health record for >1.7 million post-9/11 Veterans with DOD health records and VA care from FY2000-FY2019. Using those data, we identified Veterans with diagnoses of AD or FTD at or before the age of 65 years, which represent our early-onset AD/FTD group (n=903). These data are available in the CHOP repository. We will compare them to a group of Veterans who are at high risk of developing early-onset AD/FTD; namely, Veterans with epilepsy; moderate, severe, or penetrating [msp]TBI; or other cognitive dysfunction. We will match 50 at-risk Veterans to each Veteran with early-onset AD/FTD based on index year to account for differences in health system characteristics (eg, diagnosis and treatment) over time. Index year is the year in which they were first diagnosed with AD, FTD, epilepsy, mspTBI, or other cognitive dysfunction. As noted earlier, we have found the positive predictive value of the algorithm for identifying AD and FTD diagnoses in administrative data was 0.88‐0.96, much higher than other types of ADRD, and therefore these are the foci of this study [26,27].

Participants in the qualitative component of the study and the modified Delphi process will be recruited from among members of the LIMBIC cohort; specifically, we will invite a diverse sample of post-9/11 Veterans with early-onset AD/FTD or at high risk of ADRD to participate. If we are unable to achieve target sample sizes using this cohort alone, we will recruit post-9/11 Veterans from other ongoing studies, VA health care users, and Veterans connected with organizations related to TBI and ADRD.

### Inclusion and Exclusion Criteria

For aim 1, all post-9/11 Veterans aged 65 years or younger in the dataset will be included as long as they can be classified into one of the three groups of interest: having early-onset AD/FTD, being at high risk for ADRD in the future (Veterans with epilepsy; mspTBI; or other cognitive dysfunction), or being at lower risk of ADRD in the future (ie, not having early-onset AD/FTD, epilepsy, mspTBI, or other cognitive dysfunction). For aims 2 and 3, post-9/11 Veterans aged 65 years or younger with self-reported early-onset AD/FTD, epilepsy, mspTBI, or other cognitive dysfunction and their caregivers who are aged 18 years or older, can participate in English, and can consent to participate are eligible to do so.

To ensure that informed consent is obtained for this research, we plan to use a modified version of the University of California, San Diego Brief Assessment of Capacity to Consent (UBACC). The UBACC helps investigators identify research participants who warrant more thorough decisional capacity assessment or remediation efforts prior to enrollment [39]. Specifically, we will ask Veterans with early-onset AD/FTD, epilepsy, mspTBI, or other cognitive dysfunction four questions before they participate: (1) “What is the purpose of the study that was just described to you?”; (2) “What makes you want to consider participating in this study?”; (3) “Do you have to be in this study if you do not want to participate?”; (4) “If you participate in this study, what are some of the things that you will be asked to do?” If a potential research participant does not provide a correct response to one or more of these questions, the investigator will conduct a remediation session to re-review the relevant portions of the consent language. We will then re-administer the UBACC to potential participants to assess their understanding. If the Veteran does not provide a correct response to one or more questions, they will not be eligible to complete the study.

### Sample Size and Power

In aim 1 analyses, we expect to include 903 post-9/11 Veterans with early-onset AD/FTD (495 with early-onset AD, 303 with early-onset FTD, and 105 with early-onset AD and FTD diagnoses) and 45,150 Veterans without early-onset AD/FTD who are at high risk of developing ADRD in the future. These numbers come from the existing CHOP dataset. For aim 2, we will seek to reach saturation with the individual interviews [40,41] and anticipate interviewing up to 20 Veterans and 20 caregivers, totaling up to 40 individuals, based on existing literature [42] and our previous studies focused on HCBS and other service use among Veterans and caregivers. We will recruit only Veterans with early-onset AD/FTD or at high risk of ADRD and their caregivers since the primary purpose is to better understand their use of HCBS, barriers to this use, and preferences for HCBS that will inform future services and interventions. We intend to recruit and interview dyads—Veterans and their caregivers—but recognize that this will not always be possible and will interview only one member of the dyad if needed. Similarly, our goal in the modified Delphi process (aim 3) is to include the perspectives of Veterans and caregivers to document all possible HCBS solutions and rank them. We therefore intend to recruit a diverse sample in terms of sex, race/ethnicity, and rural residence. We plan to recruit up to 84 participants in the modified Delphi process, aiming for an equal number of Veterans and caregivers. The recommended sample size for online panels like this one is 40‐60 participants [43], and the response rates between the first and second rounds of the modified Delphi process generally appear to be around 60% [43,44]. Therefore, we anticipate that if 84 participants complete round one, we will have 50 responses in round 2.

We anticipate adequate statistical power for our planned aim 1 analyses, even if we focus on only the Veterans with EO-AD/FTD. For example, in order to detect a mean difference in service use between the Veterans with and without early-onset AD/FTD of 1.0 with a SD of 5.0% and 80% power (eg, Veterans with early-onset AD/FTD use a service 7 times, on average, and Veterans at high risk of early-onset AD/FTD use service 6 times, on average, with SD 5.0), we would need 392 Veterans with early-onset AD/FTD, assuming equal samples. Similarly, we would have 80% power to detect a 20% difference in the proportion of Veterans with and without early-onset AD/FTD using a service (eg, 25% vs 21%). These power calculations suggest that we will have adequate power to make most subgroup comparisons by sex, race/ethnicity, and geographic region for commonly used services as well. We anticipate a 25% response rate from the cohort overall for primary data collection efforts (aims 2 and 3), and a 60% completion rate in the modified Delphi process [45]. Therefore, we expect to contact 500 Veterans and caregivers to enroll 124 in the individual interviews and the modified Delphi process.

### Recruitment

For primary data collection, we will contact post-9/11 Veterans with early-onset AD/FTD or at high risk of ADRD from the CHOP repository using the contact information available in VA databases. We will engage in purposive criterion sampling to recruit female Veterans; Veterans who are African-American, other races, and Hispanic; and Veterans who live in both rural and urban areas in the study [46]. We will use a modified Dillman approach [47] for recruitment, using mailing and web-based surveys that have resulted in high participation levels in previous studies. This will include an introductory letter via mail, an electronic introductory letter via email, a second reminder about the study with details, and a final reminder with a request to participate. These introductory letters will include the purpose, the time required to participate, incentives for participating, a paper version of the preinterview or Delphi survey, and a QR code to the electronic version of the survey. Contact information for the team will also be included.

In addition to this criterion sampling recruitment approach, and if recruitment is slow, we will incorporate snowball recruitment in which prospective participants can share study information with others they know who may be eligible and interested [46]. This approach has the advantage of efficiency and, when networks are broad, final recruits can be independent of initial recruiters [48]. Similarly, since we do not have contact information for caregivers, we will rely on Veterans to share information with them. Therefore, we may have difficulty engaging these caregivers because of indirect contact. If needed, we will expand our recruitment efforts to include Military, Veteran, and Caregiver groups like the Elizabeth Dole Foundation and the Red Cross Military and Veteran Caregiver Network, among other caregiving-serving organizations. Additional recruitment strategies will include the use of community gatekeepers or leaders, especially within the caregiver community [49].

### Service Member, Veteran, and Caregiver Stakeholder Input

We will work with existing Veteran Engagement Groups established at VA medical centers to receive feedback on this project and to share findings. Veteran Engagement Groups vary by site but typically include Veterans from diverse backgrounds, including those from different service eras, military ranks, sexes, and demographic groups. Several groups also include family members or family caregivers, who will be particularly important in this study. We plan to share draft study materials, including documents and strategies related to recruitment, data collection (eg, interview guides), and dissemination (eg, framing results, lay audience summaries) with the Veteran Engagement Groups and incorporate their feedback. If needed, we will recruit additional lived experience advisors to guide us on this project to represent groups not reflected within the membership of the Veteran Engagement Groups.

### Data Collection

#### Secondary Data

LIMBIC data were derived from the CHOP repository. The VA manages the repository via the VA Informatics and Computing Infrastructure (VINCI) in a secure data warehouse. VINCI is a national resource for researchers to house data and conduct analyses with support from experts who enable the use of data securely to answer research questions and improve VA health care delivery. We have requested access to CHOP data using procedures established by the CHOP repository standard operating procedures, via VINCI’s secure environment. We have also requested VA and Centers for Medicare and Medicaid Services data to link with the existing CHOP data so that we can explore HCBS use.

We will identify HCBS use as we are currently doing for older Veterans as part of an ongoing study funded by the VA, and will use data on comorbidities, including TBI and other health care utilization such as mental health, neurology, TBI, and other specialty care that may be relevant to understand the patient context [26,50].

#### Qualitative Interview Data

The interview guide was developed to align with our conceptual framework, and it will be revised based on input from Veterans and caregivers. Our goal is to interview both the post-9/11 Veteran with early-onset AD/FTD or at high risk of ADRD and their caregiver individually [51]; however, we will allow for interviews with only one member of the dyad, depending on interest in or ability to participate, preferences, and scheduling constraints. We will also allow Veterans and caregivers to complete the interview together, if needed. We will use a descriptive qualitative study design [52] to: (1) characterize the use of HCBS since the Veteran’s diagnosis with early-onset AD/FTD or another condition that puts them at high risk of ADRD, including the types of services used, the timing of service use relative to diagnosis and any injuries/other diagnoses, the reasons for stopping and starting service use, barriers to HCBS use, promoters of HCBS use, and knowledge/awareness about HCBS availability; and (2) identify preferences for HCBS (eg, types of service, eligibility requirements, timing, delivery mode, etc). Both the Veteran’s and caregiver’s personal preferences and experiences will be detailed as part of this process. The interviews will be guided by the following research questions:

1. What unique facilitators and barriers to accessing and using HCBS do post-9/11 Veterans with early-onset AD/FTD or at high risk of ADRD and caregivers report that relate to their age, race/ethnicity, geographic residence, VA disability status, preferences for support, awareness of HCBS, and perceived usefulness of HCBS for their specific needs and situation?
2. What specific preferences do post-9/11 Veterans with early-onset AD/FTD or at high risk of ADRD and caregivers report for both the type of HCBS services and the service delivery method (eg, timing, duration, format, and location) that include aspects relating to their life stage and family needs?

The goal of this component of the study is to describe the insights and experiences of post-9/11 Veterans with early-onset AD/FTD or at high risk of ADRD and their caregivers in accessing and using HCBS. Additionally, we seek to identify important elements of HCBS that will help develop strategies for increasing appropriate utilization of these services. To collect information on demographic characteristics and baseline influences in our framework (social/economic status, family structure, cultural support systems, and Veteran health and well-being), we will ask potential participants to take a brief survey administered through Qualtrics before the interview.

The semistructured interviews will be conducted via web-based conferencing and will be audio-recorded and transcribed. Audio recording will facilitate the verbatim transcription of these interviews and the subsequent content analysis. Initial transcription will be completed using the built-in transcription function of Microsoft Teams, a secure, enterprise video-conferencing platform, and finalized by the research team in San Antonio, TX. Audio recordings and automated transcripts will be generated using Microsoft’s built-in speech-to-text service and stored within the university’s Microsoft 365 environment (OneDrive or SharePoint), which uses role-based access controls. All data are encrypted during transmission and while stored, and access is restricted to authorized members of the research team. Recordings and transcripts can be downloaded for secure storage and deleted from the cloud environment in accordance with institutional data-handling policies. While no digital platform can guarantee absolute security, Microsoft Teams complies with widely recognized data protection and security standards and is considered a low-risk platform for conducting confidential qualitative interviews. All transfers between VA and non-VA systems will follow the data use agreement established for this project. The transcripts will be the main source of information during the analysis [49]. Field notes will also be taken by the interviewer/interview team and used in analyses. These notes will include information about the interactions between Veterans and caregivers if both participants are present during the same interview. The interviewer will have training and experience in qualitative research. We estimate each interview will take approximately 60 minutes. The first two interviews will be used as pilots to assess the content and flow. Performing this pilot test will provide the opportunity to modify and add probes based on new information valuable for the aims of this study [53]. Data from the pilot interviews will be included in the final analyses.

We will use best practices in conducting qualitative data collection, management, and analysis. Interviews, including using clear and direct language to describe the study, allowing participants extra time to complete the interviews, and allowing participants to take breaks during the interview if needed, as well as an audit trail of all the study activities [51]. Participants will be compensated US $25 per completed interview (both Veterans and caregivers will be compensated individually if completing a joint interview).

#### Modified Delphi Process

The Delphi method is a structured approach designed for reaching consensus using iterative rounds of anonymous surveys that ask experts to rate various topics or ideas [54]. It has been modified over time in various ways, including an adaptation to an online format. While the Delphi process is most used to gain input from subject matter experts like clinicians or researchers, in this study, we believe the most relevant experts for informing HCBS modifications are the post-9/11 Veterans with early-onset AD/FTD or at high risk of ADRD and the caregivers themselves. The Delphi technique, especially the modified online approach, is increasingly being used to gain feedback from patients and caregivers in pursuit of developing more patient-centered care [45,55]. Combining the RAND PPMD Patient-Centeredness Method [44,45] with recent successful work by Morbey, Harding, and colleagues to engage people with dementia in a Delphi process [56,57], we plan to conduct a 2-stage online modified Delphi process for post-9/11 Veterans with early-onset AD/FTD or at high risk of ADRD and their caregivers.

The goal of the modified Delphi process is to understand priorities among Veterans with early-onset AD/FTD and their caregivers regarding potential HCBS modifications, delivery strategies, or other aspects of HCBS interventions designed specifically to meet their needs. The modified Delphi process will be guided by the following research questions:

1. What specific adaptations to existing HCBS available through VA would make them more appealing and useful to post-9/11 Veterans with early-onset AD/FTD or at high risk of ADRD and caregivers?
2. Is there consensus that several programs or approaches are important to post-9/11 Veterans with early-onset AD/FTD or at high risk of ADRD and caregivers that can be tested in future intervention studies?

The data from aims 1 and 2, along with information from the literature and our conceptual framework, will be used to develop a list of HCBS approaches that would be useful to post-9/11 Veterans with early-onset AD/FTD or at high risk of ADRD and their caregivers. This list will form the basis for the first round of surveys (Figure 2). Each potential HCBS adaptation or program will be listed using plain language and begin with the phrase, “How important is it to….” For example, items may include, “How important is it to create a program where younger Veterans can do physical exercise or projects together?” or, “How important is it to change respite so that caregivers can have a full day break on the weekend?” Participants will be asked to rate the importance of each item as “1 - Not very important,” “2 - Important,” or “3 - Very important.” This scale, while much narrower than most Delphi rating scales, which often range from 1 to 9, was developed by Morbey and colleagues in collaboration with people with dementia to make it easier for people with cognitive impairment to participate [57]. They demonstrated that it can still be used to evaluate consensus and that it was acceptable and feasible for people with cognitive impairment; furthermore, the responses from people with dementia and their caregivers varied meaningfully [57].

The survey will include text boxes to allow participants to describe why they rated each statement a certain way. Additionally, we will add a text box at the end of the survey to allow participants to identify any additional services or adaptations they would like to see that were not listed on the survey.

Once the first survey closes, responses will be summarized and provided to participants. Rankings will be summarized using bar charts to show the frequency of responses for each item. Each participant’s response will be indicated in the bar chart so they can compare their rating to other participants’ rankings. Based on the difficulty interpreting these reports among some people with dementia in the study by Morbey et al [57], we will also audio-record a summary of the overall findings from round one. While this will not be tailored to each respondent, we will send each respondent a summary report showing their own answers along with the full-sample summary report. We will summarize written comments from round one by identifying themes within responses, using a descriptive content analysis approach similar to the process described for individual interviews above. Specifically, we will code free-text responses, identify themes, validate them, and summarize themes. This will be included in the summary report along with the bar charts and individual responses.

The first round Delphi survey will be available through Qualtrics, and a unique link will be sent to each participant’s email address. The first-round survey will remain open for 4 weeks. Summary reports will be distributed within 4 weeks of the first round of the survey closing. Responses will be linked, allowing participants to recall their earlier answers in subsequent rounds of surveys. Also, linking emails to responses will enable participants with early-onset AD/FTD or at high risk of ADRD to respond over multiple sessions if they wish to do so; therefore, fatigue will be less of a barrier to participation [51].

**Figure 3.**
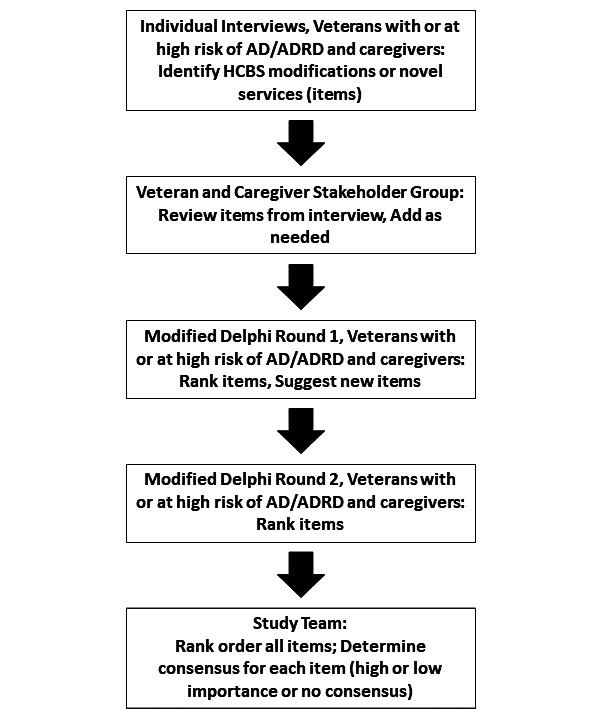
Flow diagram for primary data collection. AD: Alzheimer disease; ADRD: Alzheimer disease and related dementia; HCBS: home- and community-based service.

Once participants have received their summary reports from the first round, approximately 4 weeks after the end of the first round, the second round of the modified Delphi process will begin. The second survey round will resemble the first round but will include any newly suggested items (adaptations or new services identified in the first round). We will not collect any new HCBS-related suggestions from the second round since it is the final round in this process. When completing round 2, participants will be invited to review the summary report and consider the comments and ratings of other people, then submit their ratings for each statement. The second round of the survey will again be linked to respondents’ email addresses so they can complete it over multiple sessions if they like. The round 2 survey will close 4 weeks after it opens. The second round will form the basis of the final rankings and consensus determination (Figure 3). In our recruitment materials, we will make it clear that participants are welcome to get help from someone else when completing the surveys, though we want the opinions to be their own. Participants will receive a US $15 incentive for each round of the modified Delphi process (up to US $30 total).

### Measures

#### Diagnoses

##### Overview

We will identify AD and FTD diagnoses using inpatient and outpatient data, consistent with a previous completed study using the LIMBIC Phenotypes dataset [26]. Specifically, Veterans must have 2 or more diagnosis codes documented at least 7 days apart [20]. We define early-onset AD/FTD as AD or FTD that occurs at or before the age of 65 years. We will consider Veterans to be at higher risk of developing early-onset AD/FTD or ADRD in the future [58] if they have epilepsy, mspTBI, or another cognitive dysfunction (eg, cognitive disorder not otherwise specified, memory loss, mild cognitive impairment). TBI status and severity are classified based on ICD-9/10 codes from both DOD and VA, along with information from the comprehensive TBI evaluation, using the process established by the Chronic Effects of Neurotrauma Consortium epidemiology study and the Armed Forces Health Surveillance System [50,59,60].

##### Home- and Community-Based Services

The VA offers 11 noninstitutional programs that are delivered either in-home (homemaker home health aide, skilled care, hospice, respite, spinal cord injury and disability, home-based primary care, and telehealth) or in the community (VA, community, or state adult day care; residential care). We will use data from Geriatrics and Extended Care Data Analysis Center to classify service use within VA and will supplement this information with Medicare and Medicaid data, since Medicaid covers a substantial share of HCBS, including among VA users.

### Data Analysis

#### Aim 1: Quantify HCBS Use by Post-9/11 Veterans With Early-Onset AD/FTD or at High Risk of ADRD Using Data From VA and DOD and Compare It to Post-9/11 Veterans at Lower Risk of ADRD

We will calculate both the cumulative frequency of use (ie, number of unique service records) and the proportion of Veterans using a service (ie, binary indicator for any vs no use) among Veterans with EO-AD/FTD, Veterans at high risk for EO-AD/FTD, and Veterans at lower risk of ADRD. We will make comparisons between these groups using *t* tests for continuous measures (number of services) and chi-square tests for categorical measures (any service use) to test whether Veterans with EO-AD/FTD have higher HCBS use than other groups and whether there is variation by race and ethnicity. We will rank-order the services based on the total amount of services used and use *t* tests to compare the difference across types among Veterans with early-onset AD/FTD or at high risk of ADRD. We will conduct analyses using the crude frequency of service use and we will conduct analyses that adjust for important confounding factors based on our conceptual framework and the existing literature related to HCBS use for AD/ADRD: demographic characteristics (age, sex, race/ethnicity), rural residence, and the presence of other health conditions, namely spinal cord injury, movement disorders, and the number of chronic health conditions. We will use generalized estimating equations to calculate adjusted estimates.

We will calculate and report 95% CIs for all estimates and will use a *P* value less than .05 to indicate statistical significance in all analyses. In situations where data for a subgroup are sparse or when testing for interactions between groups, we will use a *P* value of less than .10 to indicate a statistically significant difference. Additionally, we will calculate the Cohen *d* measure of effect size, classifying results as a small effect if *d*≥0.2 and *d*<0.5, a medium effect if *d*≥0.5 and d<0.8 and a large effect size if *d*≥0.8. We will not report estimates for subgroups if they have an individual cell size smaller than 5 Veterans. We will flag estimates that have a relative standard error greater than 30.0% (Relative standard error is calculated as the standard error of the estimate divided by the point estimate, then multiplied by 100%).

#### Aim 2: Identify HCBS-Specific Facilitators, Barriers, and Preferences Among Post-9/11 Veterans With Early-Onset AD/FTD or at High Risk of ADRD and Their Caregivers

We will conduct a descriptive content analysis using Atlas.ti (version 25.0.1; Lumivero, LLC) approach for the qualitative data analysis [49,52,61,62]. Our approach will include an in-depth description of the information collected from the participants, including transcripts and field notes. We will then develop a coding system. The codes will be purposely and methodically assigned to specific statements and information. Next, we will cluster units of meaning (codes) to form themes. The data will be examined to identify common themes, extracting significant statements to compile a set of themes based on the research question, leading to the creation of meaning units and textual description. These themes will also be related back to the conceptual framework; we expect to identify factors related to Caregiver Strain/Stress and Veteran Unmet Need and Shifting Priorities and Seeking Help in particular. We will summarize each interview, creating text that incorporates all the themes elicited from the data, giving a holistic context. The summary will include verbatim examples, also known as exemplars or quotes. Finally, once all interviews have been coded and summarized, we will synthesize across interviews to create a composite summary. This final synthesis will identify points of possible intervention for policy, programs, and future research.

In addition to this descriptive content analysis, we will catalog all suggestions for HCBS adaptations or development using data from the qualitative interviews. We will consider the codes and themes identified through the process above when deciding whether to combine responses across individuals. This list of suggestions will be part of the foundation for the next step, the modified Delphi approach.

#### Aim 3: Develop and Prioritize Specific HCBS Interventions With Input From Post-9/11 Veterans With Early-Onset AD/FTD or at High Risk of ADRD and Their Caregivers

The free response data from the Delphi process will be analyzed using a descriptive content analysis approach [49,52,61], similar to aim 2. However, because this aim focuses on ranking interventions, we will not perform an exhaustive coding of the data. Instead, we will focus on identifying which aspects of HCBS the participants wanted to modify. We will catalog all HCBS adaptations and ideas identified in individual interviews, any additional ideas suggested by lived experience consultants, and ideas generated during the first round of the Delphi process. This will constitute the final list of all potential adaptations to existing HCBS available through VA that would make them more appealing and useful to post-9/11 Veterans with early-onset AD/FTD or at high risk of ADRD and caregivers.

Using the ranking data, we will determine whether consensus was reached on the importance of each potential HCBS modification or new intervention. We will calculate descriptive statistics for each item: median, mode, and interquartile range. We will use the median scores to rank the importance of individual items. To determine whether there was consensus among participants, we will calculate the proportion of people who responded “important” or “very important” for each item. If 70% or more of respondents selected these answers, we will consider there to be consensus that the item has high importance. Similarly, if 70% of respondents report an item is “Not very important” we will consider this to represent consensus that the item has low importance. All other scenarios will be classified as not reaching consensus.

### Ethical Considerations

The University of Utah (IRB #00152365), Salt Lake City VA (R&D# 152365), and the University of Texas at San Antonio (IRB #00001988) reviewed this study and classified it as exempt. The study has been reviewed by the Human Research Protection Office of the United States Army Medical Research and Development Command. All data with protected health information or personal identifiable information will remain on a server that has federally approved encryption at VA Salt Lake City. Participants must provide informed consent to participate in the interviews or the Delphi surveys. To encourage participation, we will provide an incentive of US $25 for each participant in an interview. Participant incentives for the modified Delphi process are up to US $30 for each participant (2 rounds; US $15 for each complete round).

### Dissemination

We plan to publish our findings in at least one peer-reviewed manuscript and also expect to present findings as they are generated at relevant scientific conferences. We will only present aggregated results of quantitative data, though we expect to include exemplar quotes from qualitative data in both written and oral dissemination products. When quoting participants, we will use a study identification number or a falsified name to protect their identity. We will use relevant reporting guidelines for publications, including Strengthening Results of Observational Studies (STROBE), Consolidated Criteria for Reporting Qualitative Research (COREQ), Delphi studies in social and health sciences—recommendations for an interdisciplinary standardized reporting (DELPHISTAR), Checklist for Reporting Results of Internet E-Surveys (CHERRIES), and Good Reporting of a Mixed Methods Study (GRAMMS).

## Results

### Overview

We have received ethical and data access approvals to begin the study on January 24, 2023. We met with 2 Veteran Engagement Groups, and they recommended we modify study recruitment materials and the interview guides and develop an HCBS information sheet to provide to participants. We made these changes and had the new materials approved by local, VA, and DOD ethical review boards in June 2025. We plan to begin secondary data analysis in September 2025. While secondary analysis is ongoing, we will begin primary data collection by recruiting participants for interviews beginning in October 2025. This part of the data collection will continue for 6 months. Once secondary and interview data are analyzed, we will begin recruiting for the Delphi survey, which contains 2 rounds of surveys. The project will be completed by September 30, 2027.

### Study Cohort: Secondary Data

Using the CHOP dataset, we identified 903 post-9/11 Veterans with early-onset AD/FTD (aged 65 years or younger at the time of diagnosis). We then identified 45,150 post-9/11 Veterans with epilepsy, mspTBI, or other cognitive dysfunction who were 65 years or younger at their index date and matched them 50:1 with the early-onset AD/FTD group based on the index year. The 46,053 Veterans in the preliminary aim 1 cohort averaged 55 years old at the index date and were mostly male (38,842/46,053, 84%) and non-Hispanic White (28,016/46,053, 61%). Some of these Veterans may ultimately be excluded based on study criteria or for analytic reasons (eg, missing data).

### Anticipated Results by Aim

#### Aim 1: Quantify HCBS Use by Post-9/11 Veterans With Early-Onset AD/FTD or at High Risk of ADRD Using Data From VA and DOD and Compare It to Post-9/11 Veterans at Lower Risk of ADRD

Analyses for this aim will provide us with crude and adjusted estimates of HCBS use among 2 groups of Veterans for whom these data are not currently available. Based on these results, we will identify any subgroups of Veterans who have particularly high or low service use, and we will identify factors that may be driving differences in HCBS utilization.

#### Aim 2: Identify HCBS-Specific Facilitators, Barriers, and Preferences Among Post-9/11 Veterans With Early-Onset AD/FTD or at High Risk of ADRD and Their Caregivers

Results from this aim will provide insight into the experience and processes of Veterans and caregivers seeking and using HCBS. We expect to gain more information about whether and how HCBS utilization drivers from aim 1 are indeed impacting HCBS use decisions. We also expect to identify factors not available in health systems data from these interviews, including personal, family, and social influences, and service availability. By combining the results from Aims 1 and 2, we expect to build a more comprehensive set of items for the preferences survey in aim 3.

#### Aim 3: Develop and Prioritize Specific HCBS Interventions With Input From Post-9/11 Veterans With Early-Onset AD/FTD or at High Risk of ADRD and Their Caregivers

After completing this aim, we will have a list of HCBS modifications with rankings provided by Veterans and caregivers. Furthermore, we will have identified adaptation needs for which there is consensus. These items with consensus will represent those on which we focus for follow-up with system leadership to identify opportunities to incorporate them into existing programs and services.

## Discussion

### Principal Findings

Results from this study will provide information about the current use of HCBS and identify preferences among post-9/11 veterans and their caregivers. These findings will provide tangible evidence of current HCBS use, factors associated with HCBS use, and modifications to HCBS that would make them more useful for Veterans and their caregivers. The use of long-term supports for Veterans living in the community has the potential to enable them to remain at home while limiting financial and emotional distress for their families, especially when family members are also serving as caregivers. However, if these services are not acceptable or accessible, these benefits will not be realized.

Our long-term goal is to increase HCBS utilization and effectiveness, and this study represents an approach to better understanding HCBS use to inform future efforts that will address this goal. This study allows us to use multiple modes of data collection and analytic methods to holistically assess the use of HCBS among Veterans with or at high risk of developing ADRD. We will use these results to plan for health system strategies and interventions that will improve HCBS quality and utilization. The study provides us a unique opportunity to lay the groundwork to develop evidence-based HCBS interventions for post-9/11 Veterans at high risk of developing ADRD and their caregivers that ultimately will improve quality of life and facilitate community living and participation. This study will help to develop programs based on the findings and disseminate the findings to affect policy.

### Strengths

This study incorporates numerous data sources, such as health system data from DOD, VA, and Centers for Medicare and Medicaid Services, including inpatient, outpatient, and HCBS utilization data, along with VA TBI screening from comprehensive TBI evaluation. We will leverage existing data and collect novel data to comprehensively evaluate HCBS use and potential strategies to increase uptake. The modified Delphi method we are using was developed for people living with ADRD, and this may be the first time it has been used with Veterans. While previous work has measured HCBS use among post-9/11 Veterans whose caregivers had applied to or been accepted to the VA’s Program of Comprehensive Assistance for Family Caregivers (herein referred to as the VA Caregiver Support Program) [63], no research has specifically addressed the use of HCBS among Post-9/11 Veterans whose caregivers are not enrolled in the program nor among those with or at high risk of ADRD. Similarly, we are not aware of any efforts to understand the needs and preferences among these Veterans and caregivers, which we do in this study.

### Limitations

This study may be limited in its ability to represent all geographic regions of the United States or all subgroups of Veterans. While we will aim to sample a diverse set of post-9/11 Veterans and caregivers, we will not capture the experiences of all groups, and therefore, the results may not generalize to all Veterans and caregivers. In particular, our recruitment strategy will likely result in people with diagnosis codes in VA data being more likely to be included and therefore may not reflect the experiences of Veterans who are not well-connected to VA care or to Veteran-serving organizations.

### Dissemination Plan

We plan to submit abstracts from this work at relevant scientific conferences, for example, the annual meeting of the Gerontological Society of America, the Military Health System Research Symposium, and to publish at least 2 manuscripts summarizing our findings. We will also present results within the VA health care system to the Salt Lake City Veteran Engagement Group, research and clinical colleagues via standing seminars and individual and research team meetings, and relevant operational programs, including Geriatrics and Extended Care. We will create summaries aimed at policymakers and DOD and VA staff who make decisions about HCBS funding and allocation.

### Future Directions

Results from this study will be foundational to improving HCBS availability and accessibility among post-9/11 Veterans and their families. We plan to use the findings along with feedback from Veteran Engagement Groups and operational partners within VA to identify strategies that may be acceptable to Veterans and families and feasible within the health system. We may conduct journey mapping as the next step in this process to more comprehensively document processes and implementation opportunities. We also may use the results to design and test modifications to existing HCBS programs and referral processes or create novel HCBS programs to meet the needs of Veterans and caregivers.

### Conclusions

This study will fill a critical gap to help understand the use of HCBS among post-9/11 Veterans and their caregivers and to identify strategies that could improve access to these potentially helpful services. Because VHA is an integrated and learning health care system and already provides HCBS to eligible Veterans and caregivers, it is an ideal setting in which to test modifications to HCBS design and delivery in future studies.

## Abbreviations

AD: Alzheimer disease
ADRD: Alzheimer disease and related dementia
CHERRIES: Checklist for Reporting Results of Internet E-Surveys
CHOP: Characterizing Health Outcomes in post-9/11 Veterans
COREQ: Consolidated Criteria for Reporting Qualitative Research
DELPHISTAR: Delphi studies in social and health sciences—recommendations for an interdisciplinary standardized reporting
DOD: Department of Defense
EOD: early-onset dementia
FTD: frontotemporal dementia
GRAMMS: Good Reporting of a Mixed Methods Study
HCBS: home- and community-based service
LIMBIC: Long-term Impact of Military Relevant Brain Injury Consortium
STROBE: Strengthening Results of Observational Studies
TBI: traumatic brain injury
VA: Veterans Affairs
VINCI: VA Informatics and Computing Infrastructure
